# Application of Inner Radiation Baffles in the Bridgman Process for Flattening the Temperature Profile and Controlling the Columnar Grain Structure of Directionally Solidified Ni-Based Superalloys

**DOI:** 10.3390/ma12060935

**Published:** 2019-03-21

**Authors:** Dariusz Szeliga, Waldemar Ziaja, Maciej Motyka, Krzysztof Kubiak, Jan Sieniawski

**Affiliations:** 1 Department of Materials Science, Faculty of Mechanical Engineering and Aeronautics, Rzeszow University of Technology, 12, Powstancow Warszawy Avenue, 35-959 Rzeszow, Poland; wziaja@prz.edu.pl (W.Z.); motyka@prz.edu.pl (M.M.); krkub@prz.edu.pl (K.K.); jansien@prz.edu.pl (J.S.); 2 R&D Laboratory for Aerospace Materials, 4, Zwirki i Wigury Str., 35-036 Rzeszow, Poland

**Keywords:** directional solidification, inner radiation baffle, Ni-based superalloys, Bridgman process, grain structure

## Abstract

The technique of flattening the temperature profile and controlling the formation of both the dendritic microstructure and grain structure in the directional solidification of nickel-based superalloy casting, using the novel inner radiation baffles (IRBs) in the Bridgman process, is presented in this paper. These baffles matched to the shape of mold and were placed horizontally along its height at various distances from the casting base. The plate castings of CMSX-4 superalloy were fabricated without and with the use of IRBs, withdrawing the mold at the rate of 6 mm/min from the heating to the cooling area of the industrial Bridgman furnace. Thermal analysis of the directional solidification of castings was carried out using the ProCAST software for a process where the various designs of the radiation baffle were applied. The results of the solidification conditions, the shape of liquidus and solidus isotherms, and grain structure obtained for the IRBs were compared with those reached for the standard ring-shaped (AERB) or perfectly adjusted (PARB) radiation baffles. The use of IRB resulted in flattening of the temperature distribution and decrease of the curvature of liquidus and solidus isotherms, as well as an increase of temperature gradient and cooling rate, compared with the process where AERB was only used. Consequently, primary dendrite arm spacing (PDAS) reached similar values across the width of casting and equaled to approximately 370 μm, reducing its average value by 26%, compared with the standard process. The change in predicted axial temperature gradient in casting was not found when thermophysical properties of molybdenum IRBs were used. The increase in graphite IRBs number in mold from seven to 14 caused the reduction of inhomogeneity of axial temperature gradient along the casting height.

## 1. Introduction

Ni-based superalloys are used to produce hot section components of the aircraft turbines or industrial gas turbines (IGT) [1]. These are mainly blades and vanes with conventional equiaxed (EX), columnar (DS) or single crystal (SX) structure [2,3,4,5,6]. These components are produced using investment casting by pouring the melt into the ceramic mold and then cooling at a controlled rate of the specific casting volumes [7]. The columnar or single crystal structure is formed as a result of the directional solidification process. This process is carried out by withdrawing the mold containing the melt at a specific rate from the heating to the cooling chamber of the furnace, which is separated by a thermal baffle, providing a suitable positive temperature gradient at the solidification front [8]. In the conventional Bridgman solidification process, the heat extraction from the mold surface into a water-cooled copper chill plate and chill ring proceeds by radiation. The lower part of the mold and casting are mainly cooled by intensive interaction with the chill plate. When a certain part of the alloy solidifies, the heat conduction in the direction of the chill plate is reduced, and the cooling rate of casting decreases due to the low thermal conductivity of superalloys [9,10]. However, at a certain distance from the chill plate, heat transfer becomes dominant in the radial direction to the chill ring. A ceramic mold with a complex shape, where the components are evenly distributed around the circumference, is used in the industrial cast process of directional solidification [11]. The mold surface close to the chill ring is cooled the most intensively, and the temperature gradient in the radial direction also occurs apart from the one in the axial direction. Thus, the temperature profile in the casting is uneven, and the mushy zone attains the unfavorable concave shape [12,13,14]. The curvature of the mushy zone increases with an increasing withdrawal rate of the mold when the liquidus isotherm is located in the cooling area of the furnace, especially in the large-size IGT blades. For too large curvature of the liquidus isotherm, the lateral growth of secondary dendrite arms near the casting walls can be observed which overgrow well-aligned primary dendrites [15]. Also, the shape of liquidus isotherm and value of radial temperature gradient affect the mechanism of competitive dendrite growth and the evolution of the boundary between two immediate neighbor grains with different crystal orientation. It was found that grain boundaries have the tendency to grow in a direction similar to that of heat transfer or perpendicular to the liquidus isotherm in the casting [3,9,16]. Hence, for the concave shape of liquidus isotherm, the grain boundaries will be inclined towards the middle of casting.

Besides the shape of the liquidus isotherm, thermal conditions, such as temperature gradient and cooling rate, must be strictly controlled during the directional solidification process of Ni-based superalloys [3,13,17]. Especially, the temperature gradient at the solidification front plays a key role in preventing the formation of undesirable casting defects, for example, stray grains or freckles, as well as it limits the withdrawal rate of the mold [18,19]. The increase of temperature gradient enables the growth of the withdrawal rate and reduction of production time of larger blades. Besides the economic benefits, the larger temperature gradient favorably refines the dendrite structure [10,20]. During the production of the turbine blades, one should aim for flattening the liquidus isotherm, obtaining elongated grains with the <100> growth direction and boundaries with the smallest inclination angle with respect to the withdrawal direction, simultaneously keeping the highest possible withdrawal rate of the mold [3,9,16,21].

The conventional Bridgman process has been still the most commonly used for the production of turbine blades with columnar or single crystal structure [5,8,9,13,22]. In general, in the castings produced using this process, the temperature gradient and the shape of liquidus isotherm are mainly controlled by well-known grain continuator technique [23,24,25], withdrawal rate deceleration of the mold [21,22,23,24,26,27] or using a radiation baffle placed between the heating and cooling area of the furnace [8,11]. It was found that the temperature gradient at the solidification front is the largest when the radiation baffle is perfectly adjusted to the shape of mold [11]. However, the blades have a complex shape, and their location in the mold makes it impossible to realize perfect thermal separation of the heating area from the cooling one of the furnace. Therefore, a ring-shaped radiation baffle is usually used, whose opening is matched as much as possible to the largest cross-section of the blade, providing undisturbed withdrawal of the mold from the heating to the cooling area of the furnace [8]. However, this solution does not provide the same solidification conditions on the whole cross-section of the blade because non-axial heat extraction occurs from the surface of the mold [8,12,13,28]. Therefore, the directional solidification of Ni-based superalloy using the conventional Bridgman process has been still subjected to modifications of either the baffle, mold or heaters. The application of these novel solutions, such as non-uniform mold thickness techniques [29], parallel heating and cooling method [30] or graphite chillers [28], ensures the decrease of the liquidus isotherm curvature in the platform region, and thus increases the yield and quality of castings.

Another approach to control the solidification of single crystal platform and whole blades with the use of so-called inner radiation baffles (IRBs) have been recently developed by the authors [31,32]. It was found that the modification of the mold design by placing the IRBs between the blades and the central rod significantly reduced the curvature of liquidus isotherm and increased the temperature gradient along the single crystal component compared with the standard Bridgman process [31,32]. Due to the promising results previously obtained and lack of information in the literature about this technique, an attempt was made to control the directional solidification of casting with columnar grain structure using the IRBs. The results indicate that by modifying the Bridgman process (introducing the IRBs), it is possible to obtain the solidification conditions similar to those of the process, in which the perfect thermal separation of the heating from the cooling chamber of the furnace is realized. In this way, the cooling rate increases and dendritic microstructure is more homogenized and refined at the whole cross-section of the casting. The effectiveness and usefulness of this technique for the production of the directionally solidified casting of nickel-based superalloy with columnar grains have been confirmed in this study.

## 2. Materials and Methods

### 2.1. Experimental Castings

**Figure 1:**
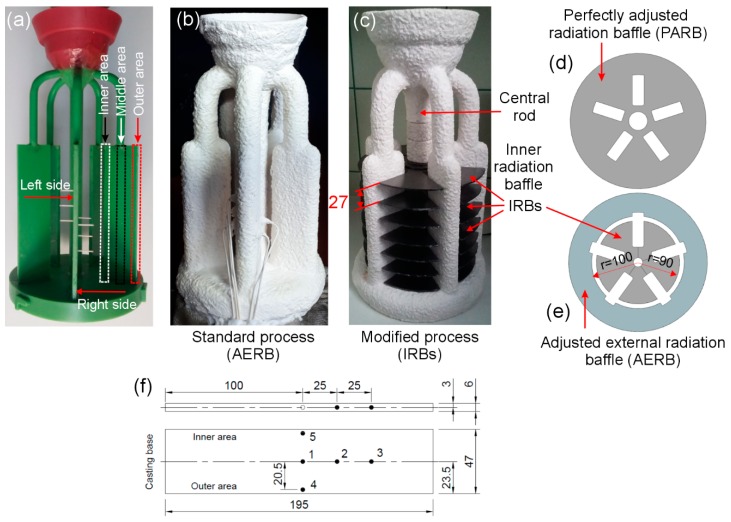
Wax model assembly (**a**); ceramic shell mold with mounted thermocouples without (**b**) and with introduced inner radiation baffles (**c**). Design of ring-shaped adjusted external radiation baffle (AERB) and inner radiation baffle (IRB) (**e**) as well as perfectly adjusted radiation baffle (**d**) which were used in experiments (**e**) and simulations (**d**,**e**). Geometric model (in mm) of plate casting with the location of measurement points (1–5) of temperature (**f**).

Melting and the directional solidification of CMSX-4 nickel-based superalloy castings were carried out using the conventional industrial scale Bridgman furnace located at the R&D Laboratory for Aerospace Materials in Rzeszow, Poland. The wax assembly consisting of five plates with dimensions of 195 mm × 47 mm × 6 mm uniformly placed on the circumference, as shown in Figure 1a, was produced. The prepared wax assemblies were the basis for manufacturing ceramic shell molds with the average wall thickness of approximately 8 mm (Figure 1b,c). Each of the molds consisted of 8 coatings. Wax assemblies were immersed into a refractory slurry and coated with refractory grains. The wax was melted out from the mold cavity using an autoclave. The residual wax and moisture were removed by burnout of the mold.

Two molds which were then used to carry out the experiments were prepared (Figure 1b,c). One standard mold was modified by placing so-called inner radiation baffles (IRBs) between the central rod and plates (Figure 1c). Seven IRBs were placed at the distance of 0, 27, 54, 81, 108, 135, and 162 mm from the mold base. Each inner radiation baffle consisted of five separate graphite elements with the same shape and thickness of 2 mm (Figure 1e). These elements were placed next to each other and then fixed in the area of the central rod creating one IRB with an external diameter of 180 mm (Figure 1e). The shape of these elements has been selected in order to provide the best matching of IRB to the external surface of the mold. One standard mold was left unmodified (Figure 1b). Five type B thermocouples with a diameter of 0.2 mm were installed in the mold (Figure 1f). The temperature was measured at three points (1, 2, 3) located along the axis of the plate at the distance of 1–100 mm, 2–125 mm, and 3–150 mm from the casting base. Additionally, the temperature measurement was conducted in the outer area (close to the heater, point 4) and the inner area (near the central rod, point 5) at the plate height of 100 mm. The method of temperature measurement with the use of uniform, bent alumina tubes was precisely explained in previous publications [9,13].

**Figure 2:**
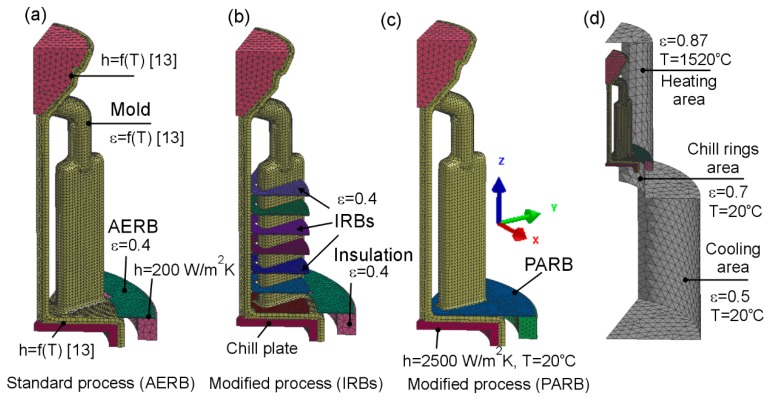
Finite elements mesh generated on model assembly for (**a**) standard process with adjusted external radiation baffle (AERB); (**b**) modified process with inner radiation baffles (IRBs); (**c**) modified process with perfect adjusted radiation baffle (PARB); (**d**) inner surface of heating and the cooling chamber of the furnace (enclosure), where h is interface heat transfer coefficient, ε is emissivity of surface.

The mold was mounted on the chill plate in the cooling chamber, and it was then moved into the heating chamber of the furnace. The ring-shaped radiation baffle (Figure 1e), with an inner diameter of 200 mm, which was adjusted to the shape of the mold, was placed on the thermal insulation plate (thickness 30 mm) of the heating chamber (Figure 2a). In the following section of the article, this part is called the adjusted external radiation baffle (AERB). The schematic image of the furnace with AERB was shown in earlier works [13,31], and, in the present study, is shown in Figure 2a,d. The mold was preheated to the temperature of 1520 °C, and then the molten CMSX-4 Ni-based superalloy of the same temperature was poured into the cavity. The directional solidification of the melt was carried out by withdrawing the mold from the heating to the cooling area of the furnace at the rate of 6 mm/min. Two experiments were conducted using the same withdrawal rate, mold and melt temperature, as well as the shape of AERB. The standard mold was used in the first experiment, while in the second experiment, the IRBs were introduced into the mold. The same AERBs were used in both experiments. In the following part of this article, the experiment, where only AERB was used, is referred to as a standard process (AERB) (Figure 1b). The experiment, in which both IRBs and AERB were used, is called as a modified process (IRBs) (Figure 1c).

The plate-shaped castings were sandblasted, and then the grains structure was revealed on the surface using etchant of following chemical composition: 2 mL HNO_3_ + 80 mL HCl + 16 g FeCl_3_ + 20 mL H_2_O. The plates were vertically placed in the container and etched at the room temperature for 15 min. After that, the castings were washed with water and alcohol and finally dried with air. Macrographs of etched plate were obtained by the digital camera. Additionally, a detailed analysis of the grain structure was conducted on the casting surface using a Zeiss Stemi 2000-C stereoscopic microscope (Zeiss, Oberkochen, Germany). For this purpose, the plate castings were cut to the height of 100 and 120 mm, and smaller elements with 20 mm height and 47 mm width were one more time sandblasted and etched (using the same process conditions as for macroscopic observation). Metallographic specimens were taken from the casting zone located close to the chill ring, symmetry axis, and central rod, which in the following section of this article are called as an outer, middle, and inner area of the plate, respectively (Figure 1a). On the plate cross-section located at the distance of 100 mm from its base, the dendritic microstructure was observed. The etching reagent used was the same as for macroscopic observation but the etching time was shorter (1–2 s). The microstructure was examined and stored using Nikon EPIPHOT 300 light microscope (Nikon, Tokyo, Japan). Primary dendrite arm spacing (PDAS) was calculated using a well-known relationship:

(1) $$\mathrm{PDAS}=\sqrt{A/N}$$

where A is analyzed area on cross-section and N is the number of dendrite cores on the surface. The value of N was in the range from 74 to 201 on the analyzed area, depending on the solidification conditions of casting. The smallest number of dendrite cores was 74 in the inner zone of casting for the standard process while the biggest one was 201 in the outer zone of casting for the solidification conditions when IRBs were used. Secondary dendrite arm spacing (SDAS) was established on the longitudinal section using the equation:

(2) SDAS=L/(n−1)

where n is the number of secondary dendrite branches on the line segment of length L. These measurements were conducted for the average number of line segments equal to 12 on each longitudinal section. Both PDAS and SDAS were determined in casting zone located in the outer, middle, and inner area of the plate.

### 2.2. Numerical Simulation

The numerical simulation of the shape of the temperature profile, thermal parameters, and directional solidification of castings was performed with the use of the macro-scale ProCAST 2015.0 software, which is widely applied to support the casting process design on an industrial scale. The geometrical models of the assembly and furnace were designed and then implemented to the software (Figure 2).

A finite element mesh was generated on the model assembly, which contained the models of chill plate, plate castings, central rod, and pouring cup. Then, the 8.5 mm-thick layer of the ceramic shell was created on the model assembly, taking into account the IRBs in the modified process (Figure 2b). These models were placed inside a geometric ambiance which describes the internal surface of the furnace consisting of the heating and cooling areas (Figure 2d). The thermal insulation plate of the heating chamber and external radiation baffle with the shape and size as in the experiment were taken into account in the proposed model. In addition, the numerical simulation of solidification was conducted for the process where the radiation baffle was used entirely separating the heating area from the cooling one of the furnace (Figure 2c). This radiation baffle contained the openings fitted to the external surface of the mold. It is called the perfectly adjusted radiation baffle (PARB) in the following part of the article. However, in the real manufacturing process of casting, it cannot be applied for practical reasons. For this process with the PARB, the solidification conditions and temperature profiles can only be determined using the numerical simulation. The influence of material and quantity of IRBs on the predicted axial temperature gradient were additionally checked (simulation 4 and 5, Table 1). Thermophysical properties of molybdenum were assumed for IRBs, instead of graphite (simulation 4). In simulation 5, the IRBs were increased from 7 to 14. The selected solidification conditions, as well as the design and size of radiation baffles, used in the numerical simulations and experiments, are given in Table 1.

Initial and boundary conditions, as well as thermophysical properties of the materials, were assumed during modeling of directional solidification. The temperature of the mold and that of the molten alloy, as well as the withdrawal rate, were assumed the same as in the experiments. The boundary condition (interface heat transfer coefficient h) and thermophysical properties of CMSX-4 Ni-based superalloy, ceramic mold, thermal insulation, radiation baffle, as well as their emissivity *ε*, were chosen based on the research conducted by Szeliga et al. [13,31] (Figure 2). These values have been carefully verified in the previous studies by comparing a large number of experimental cooling curves with the predicted ones [13,31].

The temperature profiles calculated using macro-scale ProCAST were then coupled to the meso-scale Cellular Automaton Finite Element (CAFE) model for the prediction of grain structure formation and crystallographic orientation of grains, as well as the growth kinetics of the dendrite tips, during directional solidification of the plate casting. In the current study, it was assumed that the heterogeneous nucleation of new grains of random location and crystallographic orientation took place at the casting and chill plate interface. The heterogeneous nucleation, in the CAFE model, is described by Gaussian distribution of nucleation sites according to the following equation [33,34]:

(3) $$\frac{\mathrm{dn}}{d\left(\Delta T\right)}=\frac{n_{\max}}{\Delta T_{\sigma }\cdot \sqrt{2\pi }}\exp\left[-\frac{1}{2}{\left(\frac{\Delta T-\Delta T_{m}}{\Delta T_{\sigma }}\right)}^{2}\right]$$

where ΔT is the current value of undercooling, ΔT_m_ is mean nucleation undercooling, ΔT_σ_ is standard deviation, n_max_ is the maximum density of nuclei. In this study, ΔT_m_ = 0.5 K, ΔT_σ_ = 0.1 K, n_max_ = 10^8^ m^−2^. The growth rate of dendrite tip (grains) is controlled by the total undercooling according to the Kurz-Giovanola-Trivedi (KGT) model [35], which was simplified as follows:

(4) $$v\left(\Delta T\right)=a_{2}\cdot \Delta T^{2}+a_{3}\cdot \Delta T^{3}$$

where ΔT is total undercooling of the dendrite tip, a_2_ and a_3_ are the grain growth coefficients which equal to $a_{2}$ = 3.149 × 10^−7^ m·s^−1^·K^−2^ and $a_{3}$ = 4.257 × 10^−7^ m·s^−1^·K^−3^. A detailed description of the selection of nucleation parameters and calculation of the grain growth parameters for the CMSX-4 Ni superalloy was given in [33].

## 3. Results and Discussion

### 3.1. Thermal Analysis

**Figure 3:**
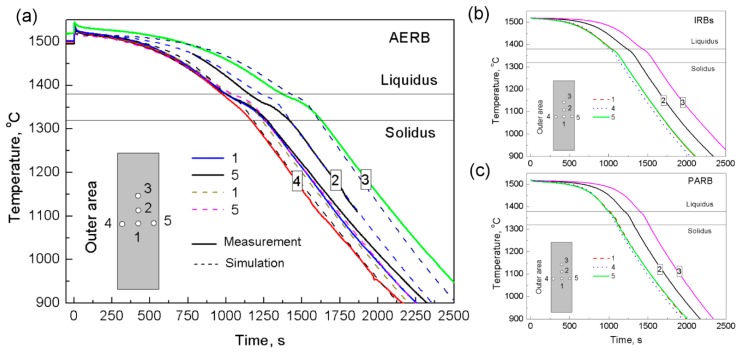
Comparison of experimental (**a**) and predicted (**a**–**c**) cooling curves at test points of casting for the process with the application of (**a**) ring-shaped adjusted external radiation baffle (AERB); (**b**) inner radiation baffles (IRBs); (**c**) perfectly adjusted radiation baffle (PARB).

**Figure 4:**
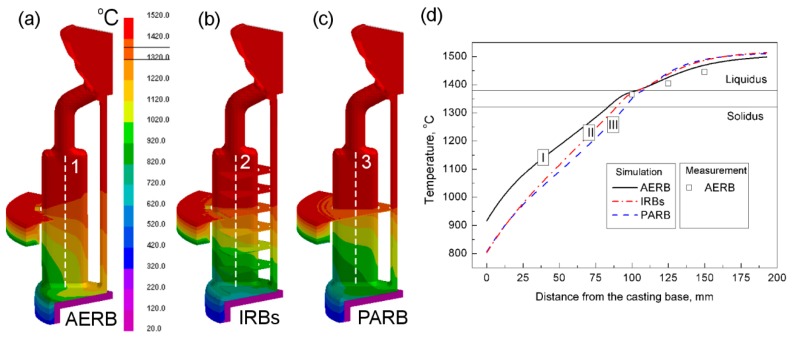
Predicted temperature field on mold surface (**a**–**c**) and temperature profiles along the symmetry axis of casting (**d**) for the process with the application of (**a**) adjusted external radiation baffle (AERB); (**b**) inner radiation baffles (IRBs); (**c**) perfectly adjusted radiation baffle (PARB). Results are presented for time 1081 s from the start of the filled mold withdrawal.

**Figure 5:**
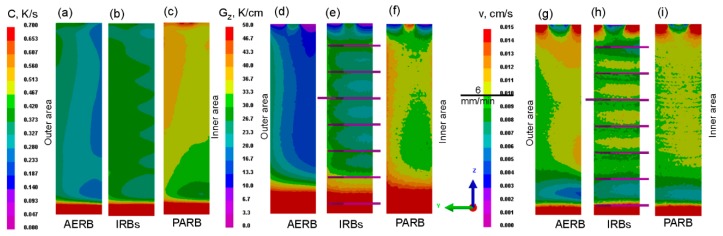
Predicted cooling rate (**a**–**c**), axial temperature gradient (**d**–**f**), and solidification rate (**g**–**i**) along the casting height. AERB: adjusted external radiation baffle; IRBs: inner radiation baffles; PARB: perfectly adjusted radiation baffle.

**Figure 6:**
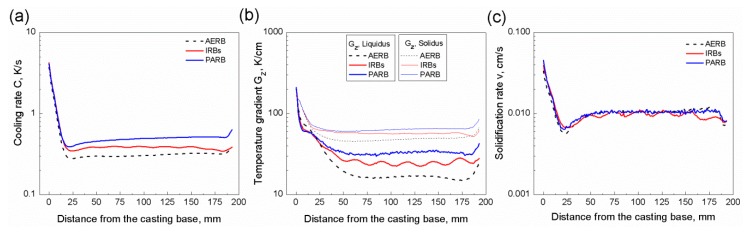
Predicted profiles of cooling rate (**a**), axial temperature gradient (**b**), and solidification rate (**c**) along the symmetry axis of the casting. AERB: adjusted external radiation baffle; IRBs: inner radiation baffles; PARB: perfectly adjusted radiation baffle.

**Figure 7:**
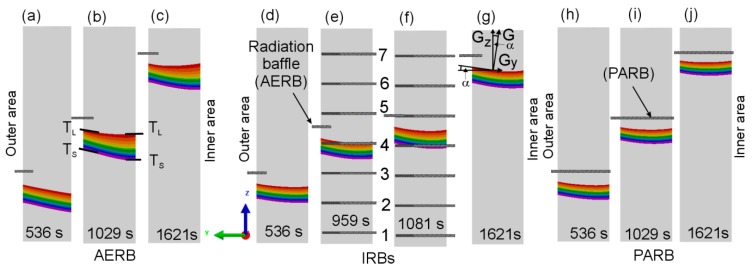
Predicted shape of the mushy zone in casting for various time points after the beginning of the filled mold withdrawal for the process with the application of (**a**–**c**) adjusted external radiation baffle (AERB); (**d**–**g**) inner radiation baffles (IRBs); (**h**–**j**) perfectly adjusted radiation baffle (PARB), where 1–7 are IRBs; Gz, Gy, and G is axial, transverse, and total temperature gradient, respectively; α is inclination angle.

**Figure 8:**
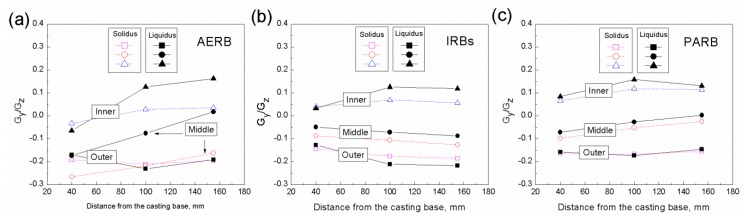
Gy/Gz ratio calculated for liquidus and solidus temperature in the outer, inner, and middle area of casting plate at a distance of 40, 100, 155 mm from its base, for the process with (**a**) adjusted external radiation baffle (AERB); (**b**) inner radiation baffles (IRBs); (**c**) perfectly adjusted radiation baffle (PARB).

**Figure 9:**
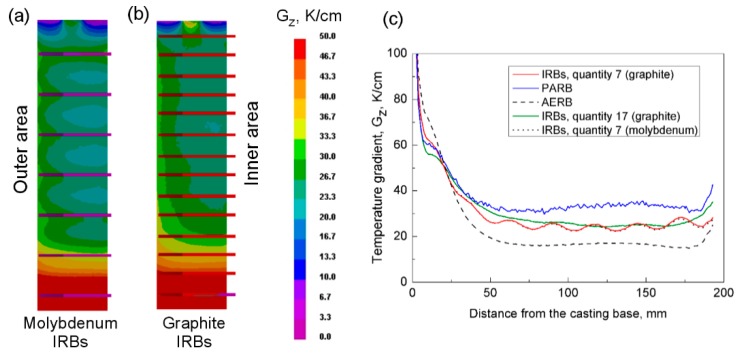
Predicted axial temperature gradient at the surface of the casting for the process with the application of seven (**a**) or fourteen (**b**) inner radiation baffles (IRBs) made of molybdenum (**a**) or graphite (**b**). Comparison of axial temperature gradient profiles for different manufacturing processes (**c**). AERB: adjusted external radiation baffle; PARB: perfectly adjusted radiation baffle.

The thermal analysis of the castings and mold were carried out in order to determine the conditions of the directional solidification process taking into account the various designs of radiation baffle (Figure 3, Figure 4, Figure 5, Figure 6, Figure 7, Figure 8 and Figure 9). The temperature measurement was performed at various points of castings which were fabricated using standard process with the ring-shaped adjusted external radiation baffle (AERB). The measured cooling curves were then compared with the predicted ones (Figure 3a). In that way, the values of thermophysical properties of the materials and boundary conditions used in numerical simulations were verified in the investigations, as well as in the previously conducted studies [9,13,31]. Good agreement of the predicted and measured temperature distribution versus time was found at points from one to five of the casting manufactured using AERB (Figure 3a). The same values of thermophysical properties and boundary conditions were used in other numerical simulations of directional solidification, in this paper, in which the inner radiation baffle or perfectly adjusted radiation baffle were introduced to the manufacturing process of the casting (Figure 3b,c).

The highest temperature gradient and cooling rate of the casting can be obtained by introducing PARB into the Bridgman furnace. Therefore, in the following part of this research, the predicted results obtained for the process with AERB and IRBs are compared with the simulations where PARB was taken into account.

The predicted temperature field at the mold surface, depending on the design of the radiation baffle, is shown in Figure 4a–c for a time equal to 1081 s from the start of the filled mold withdrawal. It was found that the temperature values changed significantly along the mold height as well as from its outer area towards the central rod for the standard process. That inhomogeneous temperature distribution between the outer and inner area of mold was caused by a shadow effect (Figure 4a) [13,28,32,36]. The radiation heat transfer between the heater surface and the mold surface (being close to the central rod) was reduced due to the shadowing caused by the neighboring plate castings (Figure 4a). The increase of surface area of the radiation baffle by using the IRBs or PARB resulted in better shielding and separation of the heating area (heater) from the cooling area (chill ring) of the furnace. Thus, the temperature increased in the inner area relative to its value in the outer area of the mold, and the shadow effect lowered (Figure 4b,c). In that way, the temperature distribution across the width of mold and in its part above the IRB or PARB was more homogeneous compared with the standard process.

The temperature distribution in the mold also influenced the heat transfer through the casting and determined the shape of the mushy zone [3,13,15]. Therefore, in order to establish the intensity of thermal impact of heater or chill rings on the mold, the temperature distributions were determined in the outer (point 4), middle (point 1), and inner (point 5) area of the casting at the height of 100 mm from its base, depending on design of radiation baffle (Figure 3). When the melt was above the radiation baffle (heating area), its volume, being near the heater, reached the higher temperature (curve 4) than in the inner area (curve 5) (Figure 3a). However, below the radiation baffle, the solidified casting close to the chill ring (curve 4) was intensively cooled and attained the lowest temperature value. In the middle and inner area of the casting, the temperature was similar (curves 1 and 5) independently of the baffle design used. The temperature difference between the outer (curve 4) and inner (curve 5) area decreased significantly when the IRB or PARB was introduced to the process (Figure 3b,c). 

The predicted temperature profile along the height of the casting symmetry axis was also determined after a time equal to 1081 s from the start of the filled mold withdrawal (Figure 4d). Change of this temperature ΔT at height Δz affects axial temperature gradient Gz at the liquidus isotherm according to the following formula [9]:

Gz = ΔT/Δz(5)

Therefore, the temperature difference ΔT should be as high as possible in order to maximize Gz. It is possible to be realized, for example, by increasing the temperature of the heaters [13]. In this study, the melt temperature, above the liquidus isotherm, was the highest when IRBs or PARB were used (Figure 4d; curves II, III). However, below the liquidus isotherm (in the mushy zone), temperature decreased significantly compared with the standard process (curve I) where only AERB was used. For the modified process with the use of IRBs, the temperature remained higher (curve II) than in the casting fabricated using PARB (curve III). Temperature difference ΔT and temperature gradient Gz are the highest when the shape of openings in the baffle is perfectly fitted to the outer surface of the mold [11]. Consequently, the heater has not got a thermal impact on the mold surface below the baffle to a greater extent. In this case, the chill ring and additionally chill plate have a thermal influence on the mold surface, providing the reduction of shadow effect. However, such a perfect shielding, between the heater and cooling area, by introducing the radiation baffle is not possible due to the complex shape of the blade geometry and the design of the mold [11]. IRBs, introduced to the furnace, more effectively separated the heating area from the cooling area of the furnace than the AERB. Hence, below the solidification front, the temperature was also lower compared with the standard process (Figure 4d). 

At a distance greater than 25 mm from the chill plate, the temperature increased in the casting with IRBs (curve II), relative to its value where PARB was used (curve III) (Figure 4d). The IRBs, located below the AERB (Figure 4b) in the cooling area of the furnace, can create the radiation shields, reducing the heat extraction by radiation from the mold area (at the position of the mushy zone) to the chill plate. Each of the consecutive IRBs increases the thermal resistance in the radiation path [37]. Therefore, in the process with IRBs, the cooling of the mold is mainly realized by the radiation heat transfer from its surface to the surface of the chill rings, while the thermal impact of the chill plate can be neglected at a certain height of the casting. Hence, below the solidification front, the temperature was also higher than in the casting with PARB (Figure 4d). On the other hand, IRBs located above the solidification front can effectively prevent heat loss from the heating chamber, through the gaps between the castings, to the cooling chamber. Due to the application of IRBs or PARB, the thermal impact of the heater on the bottom surface of the mold decreased, especially in its inner area located close to the central rod, resulting in homogeneous temperature distribution across the width of casting (Figure 4a–c).

The predicted temperature distribution was used as a basis for determining the solidification parameters, such as cooling rate, the temperature gradient at liquidus and solidus isotherms, as well as solidification rate in the castings, which were produced using AERB, IRBs or PARB (Figure 5 and Figure 6). Figure 6 shows the profiles of these solidification parameters along the height of the casting. These parameters reached the lowest values in the bottom part of the casting, in the area of contact between the melt and chill plate, as expected. With increasing distance from the casting base, these values decreased significantly, and at the height of 20 mm, the cooling rate and solidification rate attained the lowest values. In the height range from approximately 45 mm to 170 mm, they changed slightly, and the steady-state solidification conditions (SSSC) were formed as it was also reported in [9]. In this range, the solidification rate reached a similar value to the mold withdrawal rate of 6 mm/min (Figure 6c). It was found that for the standard manufacturing process, the lowest average values of cooling rate and the axial temperature gradient of approximately 0.3 K/s and 16 K/cm were achieved, respectively, in the SSSC area. The application of IRBs resulted in an increase of these parameters to approximately C = 0.4 K/s and Gz = 25 K/cm. As expected, the highest axial temperature gradient and cooling rate of 0.5 K/s and 33 K/cm, respectively, were reached for the process with the use of PARB (Figure 5 and Figure 6).

The predicted profiles of the axial temperature gradient at the solidus isotherm were also determined along the casting height (Figure 6b). It was the highest in the area of contact between the melt and chill plate, and it was then reduced significantly until the stabilization was obtained, for the range of casting height from 25 to 175 mm. This value was much higher than the temperature gradient at the liquidus isotherm and was approximately 47, 56, and 63 K/cm for the process with AERB, IRBs, and PARB, respectively. 

It was found that the solidification parameters also changed across the width of the casting, attaining various values in its internal and external areas (Figure 5). The cooling rate and axial temperature gradient were the highest in the outer area located close to the chill ring. Their values decreased toward the inner area, especially for the process in which only AERB was used. For the modified process, the cooling rate and temperature gradient changed to a lesser extent from the outer area towards the inner one, compared with the standard process. 

Based on the analysis of the profiles shown in Figure 6, it was concluded that the inhomogeneity of solidification rate and axial temperature gradient occurred along the height of the casting manufactured using IRBs, while they reduced if only one baffle was used (Figure 6b,c). In this modified process, the IRBs were continuously moved relative to AERB which was placed on the thermal insulating plate (Figure 2) of the heating chamber. The relationships between the position of IRBs (relative to AERB) and both the axial temperature gradient and solidification rate were established (Figure 5e,h and Figure 7e,f). The highest temperature gradient occurred locally at selected regions of the casting which correspond to the location of IRBs (Figure 5e). When the liquidus isotherm was shifted to the position of IRB no. 4 (Figure 7e), the solidification rate decreased locally to its smallest value (Figure 5h). At that time, the AERB was between IRB no. 4 and 5 (Figure 7e). Hence, it was found that the maximum temperature gradient corresponded to the minimum solidification rate (Figure 6b,c), as it was also reported in [13]. When the mold was withdrawn to the next level (Figure 7f), the liquidus isotherm moved towards the IRB no. 5. For the time when IRB no. 5 was at the same position as AERB, the gap between them was the smallest (Figure 7f). For such a position of the liquidus isotherm, the mold part, which was below IRBs, was most effectively cooled by the chill ring (Figure 5b). Consequently, the liquidus isotherm shifted toward the position of IRB no. 5 at an increased rate. The height of the mushy zone increased as well (Figure 7f). 

The predicted shape and height of the mushy zone at a different distance from the casting base were determined (Figure 7). It was found that the shape of liquidus and solidus isotherms changed depending on their distance from the casting base. For the process with AERB, the liquidus isotherm at the plate height of approximately 40 mm was inclined towards the inner area of the casting (Figure 7a). However, for a larger distance from the casting base, the shape of liquidus isotherm was concave while the solidus isotherm was inclined (Figure 7b,c). Hence, the height of the mushy zone unfavorably increased in the inner area of the casting. The use of IRBs in the process resulted in maintaining a similar height of the mushy zone across the width of the casting as well as along its height (Figure 7d–g). For the process with PARB, the height of the mushy zone was the smallest (Figure 7h–j), while its shape was similar to the one when IRBs were applied.

The degree of curvature of the liquidus or solidus isotherms is often characterized by the inclination angle α (Figure 7g) [15]. It is defined as the angle between the tangent to the curved isotherm and the horizontal. The shape of the liquidus and solidus isotherms, and hence the inclination angle, is directly associated with the predicted local temperature gradients in the axial Gz and transverse Gy directions according to the following relationship [20]:

(6) $$\mathrm{tg}\alpha =G_{y}/G_{z}$$

Hence, the Gy/Gz ratio also corresponds to the isotherm curvature. In this study, the Gy/Gz ratio was determined at three points evenly spaced across the casting width, in its outer, inner, and middle area (Figure 8). The axial temperature gradient was calculated for the temperature equal to 1320 °C and 1382 °C at the distance of 40, 100, and 155 mm from the casting base. When the transverse temperature gradient was 0 K/cm, then α lowered to 0° and the liquidus isotherm attained a flat shape. Consequently, Gz was equal G and the axial heat transfer occurred. The inclination angle increased with the increasing value of Gy/Gz ratio. It was found that the value of Gy/Gz ratio for liquidus and solidus temperatures varied in a small range along the height of casting fabricated using IRBs or PARB (Figure 8b,c), unlike the process where AERB was used (Figure 8a). For the modified process, the Gy/Gz ratio was similar for the liquidus and solidus temperatures as well (Figure 8b,c). Hence, these isotherms reached identical shape along the whole casting height (Figure 7d–j).

It was found that the Gy/Gz ratio for the liquidus temperature reached the lowest value in the middle area of the plate and it increased towards the edge of the plate, regardless of the baffle design or conducted process. The Gy/Gz ratio and inclination angle were higher in the outer area than in the inner one of the casting for all used radiation baffle types. It should be noted that despite the use of PARB, the flat shape of the liquidus and solidus isotherms was not fully obtained (Figure 7h–j). That showed that the non-axial thermal conditions still occurred along the casting height for such a design of the baffle and the mold.

The analysis of the shape of liquidus and solidus isotherms, as well as the Gy/Gz ratio, confirmed that nearly the steady-state thermal conditions were attained along the casting height for the process with IRBs, similar to the conditions of the process with the perfectly adjusted radiation baffle. The unfavorable significant change in the inclination of isotherms, especially in the lower part of the casting, was not found in spite of those observed in the process with AERB.

Radiation baffle component is usually made of graphite sheets [16] because graphite is characterized by high melting point and relatively low thermal conductivity; additionally, it can be quite easily machined—compared to the metals. Besides graphite, tungsten or molybdenum seems to be good sheet components of radiation baffle because of their low surface emissivity [38]. However, their application in directional solidification of turbine blades was not confirmed in publications yet. Whereas zirconium thermal baffle was already used as reported in references [3,38]. Therefore, it is important to determine the effect of IRBs material on the temperature gradient (Figure 9a). However, IRB should be thin for easy mounting in the mold and should be characterized by relatively good machinability. Hence, molybdenum IRB was taken for the analysis, while zirconium was definitely excluded. The value ε = 0.2 of surface emissivity was employed [39] and required thermophysical properties, as the function of temperature was taken from the reference [40]. Exemplary, thermal conductivity, specific heat, and density of molybdenum at the temperature of 1227 °C are λ = 98 W/(m·K), c_p_ = 0.33 kJ/(kg·K), d = 10220 kg/m^3^, respectively. The temperature gradient in castings with graphite IRBs was compared with the value obtained for molybdenum ones (Figure 9c). It was found that the application of IRBs made of Mo did not cause a noticeable increase in the temperature gradient along the casting height.

It was found that spacing of IRBs at the distance of 27 mm from each other in the mold caused inhomogeneity of solidification rate and axial temperature gradient along the casting height (Figure 6b,c). The highest temperature gradient occurred locally at the place of IRB mounting. Hence, a further modification of the mold was performed. Insertion of seven additional baffles caused the reduction of the distance between IRBs to 12.5 mm (Figure 9b). Based on the numerical simulation, it was found that such modification positively reduced local inhomogeneity of axial temperature gradient along the height of casting and simultaneously increased its value up to about 3 K/cm (Figure 9c). However, application of too little distance between IRBs can make considerably difficult their assembling in mold and complicate mold preparation. Hence, it was stated that the distance of 27 mm between IRBs is justified practically.

### 3.2. Dendritic Microstructure

**Figure 10:**
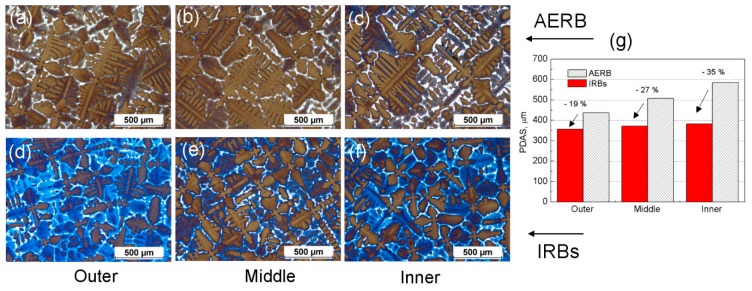
Example of dendritic microstructure (**a**–**f**) and primary dendrite arm spacing (PDAS) values (**g**) in outer (**a**,**d**), middle (**b**,**e**) and inner (**c**,**f**) area of cross-section placed at casting height of 100 mm for the process with use of adjusted external radiation baffle (AERB) (**a**–**c**) and inner radiation baffles (IRBs) (**d**–**f**).

**Figure 11:**
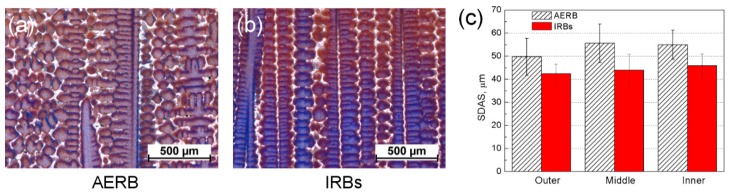
Example of the dendritic microstructure (**a**,**b**) in middle area of casting and secondary dendrite arm spacing (SDAS) values (**c**) at height 100 mm for the process with the use of adjusted external radiation baffle (AERB) (**a**) and inner radiation baffles (IRBs) (**b**).

The dendritic microstructure of castings manufactured using AERB and IRBs was examined (Figure 10 and Figure 11). Primary dendrite arm spacing (PDAS) and secondary dendrite arm spacing (SDAS) were determined in the outer, middle, and inner areas at the transverse (Figure 10) and longitudinal (Figure 11) sections of casting. The measurements were carried out at the distance of 100 mm from the casting base, where the steady-state solidification conditions occurred (Figure 6). It was found that the distribution of PDAS changed across the width of the casting, depending on the distance of the analyzed area from the chill rings and the baffle design used. In the inner area, close to the central rod, PDAS reached its largest value 585 μm, and it decreased towards the chill rings for the standard process (Figure 10). The use of IRBs reduced PDAS significantly, reaching a similar value across the width of the casting equal to approximately 370 μm (Figure 10d–f). The PDAS lowered by nearly 35% and 19% in the inner and outer area, respectively, compared with the standard process.

On the basis of thermal analysis and results of microstructure examination, the relationship between the PDAS and the temperature gradient, as well as the solidification rate, were confirmed according to the well-known equation [41]:

(7) $$\mathrm{PDAS}=K_{1}G^{-1/2}v^{-1/4}$$

where K_1_ is material constant, Gz is an axial temperature gradient, and v is solidification rate. Assuming that the solidification rate was similar in both modified and standard processes (Figure 6c), the PDAS mainly depended on the axial temperature gradient. Therefore, in the castings manufactured using IRBs, the PDAS was lower compared with its value obtained in the standard process. In addition, a correlation between PDAS (Figure 10) and mushy zone height H (Figure 7) could be observed. The average height also depends on the value of the axial temperature gradient Gz in the mushy zone:

(8) $$H=\Delta T_{L-S}/G_{Z}$$

where ΔT_L−S_ is the difference between liquidus and solidus temperature. Therefore, PDAS would decrease with the reduction of mushy zone height. The relationship between the measured PDAS and the predicted height of the mushy zone could also be determined across the width of the casting. The PDAS value for the process with IRBs varied in a small range over the entire cross-section of the casting (Figure 10d–f), in the same way as the height of mushy zone (Figure 7d–g) despite the curvature of liquidus and solidus isotherms. However, for the standard process, the PDAS and H increased gradually from the inner area towards the outer area of casting (Figure 7a–c and Figure 10a–c). Based on the analysis of dendritic microstructure, the results of numerical simulation of thermal conditions, in different casting areas, could also be approximately verified. The predicted mushy zone height is in reasonable agreement with the change of PDAS in this investigation.

The average value of the SDAS over the entire cross-section of the casting was approximately 55 μm and 43 μm for the Bridgman process with the use of AERB and IRBs, respectively (Figure 11). The SDAS reached the smallest value in the outer area, close to the chill rings, and then increased towards the inner area independently of the baffle design used. It was found that the SDAS values changed in a different way than the PDAS in particular casting areas. That effect was the most noticeable in the casting of the standard process, where in the inner area, the SDAS was similar to the value in the middle area (Figure 11c), unlike for the PDAS. The PDAS increased gradually towards the central rod. The relationship between the SDAS and cooling rate was analyzed, and it was found that the change of SDAS values over the entire cross-section was dependent more on cooling rate (Figure 3 and Figure 5a) than on the temperature gradient. These results agree with a well-known relationship [42]:

(9) $$\mathrm{SDAS}=K_{2}C^{-1/3}$$

where K_2_ is material constant, C is the average cooling rate. Therefore, SDAS decreases with increasing cooling rate. 

The experimental results of the PDAS obtained in the current studies were compared with the values presented previously in the literature for the LMC [16,20] (Liquid Metal Cooling), GCC [43] (Gas Cooling Casting), DWDS [44,45] (Downward Directional Solidification), and FCBC [46] (Fluidized Carbon Bed Cooling) processes (Table 2). The results of PDAS in this analysis were mainly adopted for the dummy blades which were manufactured with the use of both the complex mold geometries and industrial scale furnace. The average PDAS value was about 500 μm and 365 μm for the standard Bridgman and modified process, respectively (experiments 1–6). For other processes, the PDAS reached approximately 275 μm (LMC, experiments 8, 10), 320 μm (GCC, experiment 12), 275 μm (DWDS, experiments 14, 16), and 300 μm (FCBC, experiment 18). The effectiveness of a given process was assessed on the basis of the refinement of the dendritic microstructure. The PDAS values obtained for the standard Bridgman process were compared with those achieved using other methods, and the percentage of reduction in the PDAS was calculated. It was found that the use of IRBs resulted in a reduction of the PDAS approximately by 26% at the withdrawal rate of 5 and 6 mm/min (experiments 1–5). The refinement of dendrite microstructure for this technique was much higher than when only the wall thickness of the mold was taken into consideration in the standard Bridgman process (experiment 19). The decrease of the wall thickness by 32.3% led to the reduction of PDAS by 8% [47]. In the dummy blades solidified using the DWDS process at the withdrawal rate of 2.5 mm/min, the PDAS decreased by 33% (experiments 13, 14). In these experiments, the withdrawal rate was assumed the same in the conventional Bridgman process and in the improved processes. The analysis of the obtained results showed that the refinement of the dendritic microstructure increased with an increasing withdrawal rate of the mold for the LMC, DWDS, and FCBC processes (experiments 7–12, 17, 18) [16,20,46]. However, for the process with IRBs, the PDAS reduced approximately by 14% compared with a lower withdrawal rate of 3 mm/min (experiments 5, 6). The results presented in Table 2 allowed the approximate comparison of the effectiveness of dendritic microstructure refinement in the analyzed processes, because different withdrawal rate, the design of casting and mold, as well as heater temperature, than those in the current study were applied.

Generally, the IRBs can be applied in the manufacturing process of complex shaped blade castings made of Ni- or Co-based superalloys, instead of standard ring-shaped baffles. Due to the complex shape of the mold, currently used standard ring-shaped baffle can be only partially fitted to the biggest cross-section of the blade. Therefore, heat loss occurring through the gap between casting and central rod increases the curvature of liquidus isotherm. It is believed that the efficiency of IRBs in the directional solidification process is getting greater for the bigger size of chill plate and castings and higher shape complexity causing a bigger size of the gap. Based on literature data, it was found that single crystal components made of such materials like silicon, gallium arsenide, and sapphire usually are in the shape of the cylinder located in the center of the furnace [48]. Such location in the furnace and their simple shape enable the use of ring-shaped single-hole baffles, with holes that can be maximally fitted to the crucible shape. Therefore, application of AERB instead of IRBs is purposeful for manufacturing simple shaped elements made of materials other than Ni-based superalloys.

### 3.3. Structure of Grains

**Figure 12:**
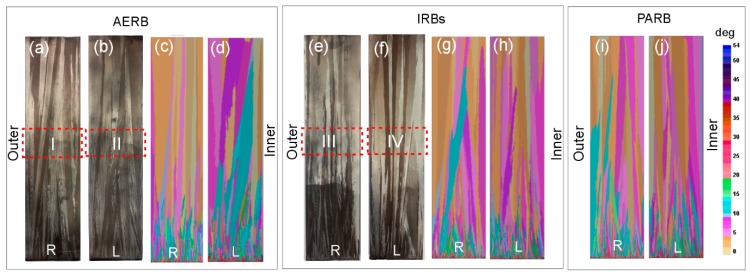
Real (**a**,**b**,**e**,**f**) and predicted (**c**,**d**,**g**–**j**) grain structure at left (L) and right (R) side of casting surface along its height for the process without (**a**–**d**) or with the use of inner radiation baffles (IRBs) (**e**–**h**) or perfectly adjusted radiation baffle (PARB) (**i**,**j**), where I–IV are areas of detailed analysis of grain and dendrite microstructure. AERB: adjusted external radiation baffle.

**Figure 13:**
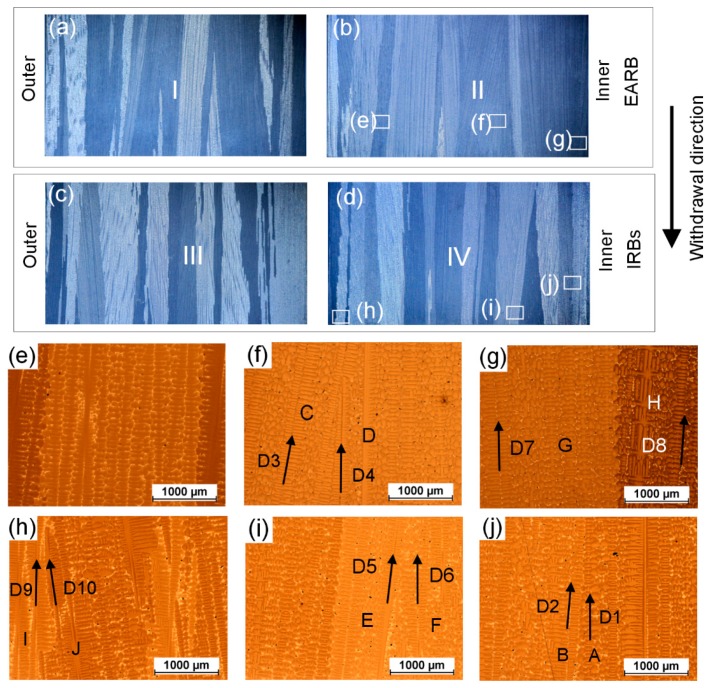
Grains structure at left (**b**,**d**) and right (**a**,**c**) side of casting surface at a height ranging from 100 to 120 mm for the process without (**a**,**b**) and with use of inner radiation baffles (IRBs) (**c**,**d**). Example of dendritic microstructures at selected areas (**e**–**j**) of casting surface—the direction of dendrite trunk growth (D1–D10) is schematically marked with arrows in grains.

The real grain structure was analyzed at the external surface of both left and right sides of plate castings after the directional solidification process without and with the use of IRBs (Figure 12a,b,e,f). The grain structure and crystallographic orientation of grains were predicted using the CAFE model for the experimental manufacturing processes (AERB, IRBs) and additionally for the process with the use of PARB (Figure 12c,d,g–j). The CMSX-4 Ni-based superalloy consists of two main phases, such as the γ phase and γ′ intermetallic phase (Ni_3_Al), with a face-centered cubic lattice [1,44]. The growth direction of the dendrite trunks (dendritic grains) coincides with <100> crystallographic direction in the new nucleus (grains) nucleated at the surface of the chill plate [5,12,33,34,42]. The mechanical properties of directionally solidified Ni-based superalloys are dependent on the crystallographic orientation of grains. Therefore, the preferential <001> growth direction of grains should be parallel to the casting axis [3,16,21]. In this study, the crystallographic orientation of grains was defined as deviation angle between <001> direction of the grain and casting axis which coincides with withdrawal direction of the mold. It is represented by different colors at the surface of the casting. The simulated grain structures were compared with experimental macrographs of the plate casting obtained after etching, and good agreement was found (Figure 12). In both the experiments and simulations, typical elongated columnar grains along the casting height were detected, independently of the process carried out. The number of these grains decreased while their transverse size increased with increasing distance from the chill plate, due to the mechanism of competitive dendrite growth, similar to the other presented investigations [3,4,5,12,16,22,33,34]. However, in the outer area of casting, the grain boundaries were inclined mainly towards the plate axis in both the simulation and experiments for the standard process without IRBs (Figure 12a–d and Figure 13a,b). Thus, in that analyzed area, their transverse size also increased especially in the upper part of the plate. As can be seen, the boundaries in the inner area were better oriented than in the outer one. It should be emphasized that the boundaries and columnar grain should be oriented parallel to the turbine blade axis as close as possible [3,16,21]. The application of IRBs or PARB in the manufacturing process ensured the reduction of grain boundary inclination towards the center of the plate (Figure 12e–j and Figure 13c,d). The boundaries were better oriented, and the grains seemed to grow more parallel to the axis of the plate, especially in its outer area.

The formation of grain boundaries and the crystallographic orientation of grains in the castings depends mainly on the ability to have a competitive grain overgrowth and the heat flow direction. This direction is consistent with the direction of the total temperature gradient. The mechanism of competitive grain growth and boundary formation in the directionally solidified castings is well-known and have already been described in many studies [9,49,50,51]. The most accepted model of competitive dendritic growth (also called competitive grain growth) was described by Walton and Chalmers [49]. In this model, unfavorably oriented dendrites are blocked by favorably oriented ones, which grow with respect to the total temperature gradient or heat flow direction.

The examples of converging (Figure 13h,j) and diverging (Figure 13g) dendrite growth can be seen in Figure 13 as in the Walton and Chalmers model. For the converging growth (Figure 13j), some of the dendrite tips (D2) from grain B were stopped on the lateral surface of grain A (D1). In the case of diverging growth, favorably oriented dendrites arms D7 from grain G overgrew unfavorably oriented dendrites D8 from the neighboring grain H (Figure 13g). However, it can also be seen, that the presented results of grain formation, in both cases of diverging and converging dendrite growth, differ from those achieved in the model proposed by Walton and Chalmers (Figure 13f,i). It was found, that for the converging growth, the unfavorably oriented dendrites (D3 and D5) were able to overgrow the favorably oriented ones (D4 and D6) as shown in Figure 13f,i. Zhou et al. [50] also observed such a phenomenon. 

As previously concluded, the value of Gy/Gz ratio and thus the shape of the liquidus isotherm may have also an important effect on the morphology of columnar grains. On the basis of the analysis of heat flow and macrostructure of the castings, it was established that a noticeable relationship occurred between the shape of liquidus isotherms and grain boundary formation (Figure 7 and Figure 12). Therefore, for the process without IRBs (Figure 12a–d), the greatest inclination of grain boundaries towards the casting axis was observed, especially in the outer area of the plate at the distance of 40 mm from its base. By applying the IRBs, it can be possible to reduce the curvature of liquidus isotherm and improve the grain structure quality (Figure 12e–h), to the level similar to that obtained in the castings solidified using a perfectly adjusted radiation baffle (Figure 12i,j). The inclination of the grain boundaries to the casting center decreased and grains became more elongated when the lateral heat flow decreased.

The effect of the inclination of heat flow direction on the competitive grain growth can be seen in Figure 13h, where another case was considered. For the converging growth (Figure 13d,h), dendrites D9 with a smaller inclination to the casting edge were more favorably oriented relative to the heat flow direction (Figure 7e) than dendrites D10. Hence, the favorably oriented dendrites D9 from grain I gradually blocked the growth of misaligned dendrite tips D10 from grain J, creating a grain boundary along the height of the casting, slightly inclined towards the casting axis (Figure 13d), thus to the heat flow direction. When the heat flow direction becomes parallel to the casting axis (for the above case), the grain boundary should be also aligned to the plane of dendrites D9, or it will be even inclined towards the outer region.

**Figure 14:**
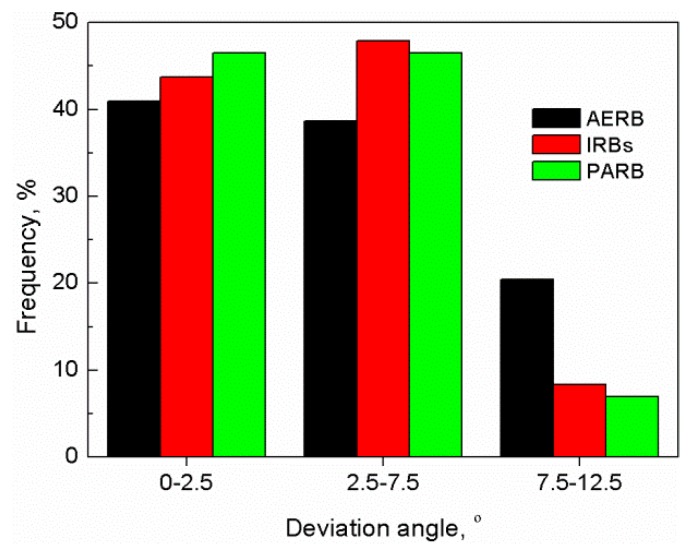
Predicted distributions of deviation angle between <001> direction of grains and withdrawal direction of mold at casting height of 100 mm. AERB: adjusted external radiation baffle; IRBs: inner radiation baffles; PARB: perfectly adjusted radiation baffle.

The predicted deviation angle between the <001> direction of the grains and withdrawal direction of the mold was determined at the casting height of 100 mm (Figure 14) depending on the design of the radiation baffle. The average deviation angle was less than 12.5° at that casting height for three baffle designs. However, the largest number of grains with the highest deviation angle in the range of 7.5°–12.5° was found in the casting produced using the standard Bridgman method. In contrast to the standard process, the number of unfavorable oriented grains (with the deviation angle in the range of 7.5°–12.5°) decreased in the casting solidified using IRBs or PARB, indicating that such baffle designs can be applied to improve the grain structure quality.

## 4. Conclusions

The manufacturing process of directionally solidified castings of CMSX-4 nickel-based superalloy, using industrial Bridgman furnace with inner radiation baffles (IRBs) applied along the mold, was developed and the results were presented in this paper. Based on the results obtained, the following conclusions were drawn:

1. For the process with IRBs, the temperature profile across the width of the casting plate and mold became more flattened than in the casting produced using only the standard ring-shaped adjusted radiation baffle (AERB). By the application of IRBs, the shadow effect in the inner area of the casting and mold was significantly reduced. Hence, the mushy zone reached a smaller curvature and attained a similar shape along the entire casting height.
2. The steady-state solidification conditions developed at a distance from the casting base ranged approximately from 45 to 170 mm. At the casting height of 100 mm, the cooling rate and axial temperature gradient increased favorably from approximately 0.3 K/s and 16 K/cm to 0.4 K/s and 25 K/cm for the process without and with the use of IRBs, respectively.
3. Both the PDAS and SDAS decreased at the casting height of 100 mm due to an increase in axial temperature gradient and cooling rate. The PDAS was similar over the entire cross-section of the casting and reached approximately 370 μm, the value lowered even by 35% in the inner area of the plate, compared with the standard process. The average value of the SDAS equaled approximately 55 μm and 43 μm for the process with the use of AERB and IRBs, respectively. Consequently, the dendritic microstructure was more homogenized and favorably refined across the width of the cross-section of the casting.
4. For the process with IRBs, it was possible to reach the solidification conditions and grain structure similar to those obtained in the manufacturing process with the application of the perfectly adjusted radiation baffle (PARB). In these castings, the grains are more elongated, and both their crystallographic orientation and inclination of boundaries towards withdrawal direction of the mold can be reduced, especially in the outer area of the plate.
5. Employment of molybdenum IRBs thermophysical properties in simulation did not cause a significant change of temperature gradient along the casting height, compared to the process using graphite IRBs. The increase of graphite IRBs amount in mold from seven to 14 reduced the inhomogeneity of the axial temperature gradient along the casting height.

## Figures and Tables

**Table 1:** Process conditions for experiments (E) and numerical simulations (P) conducted.

Experiment (E) or Simulation (P) Designation	Inner Radiation Baffle	External Radiation Baffle	Process Designation	Solidification Process Conditions
1 (E, P)	-	AERB, d = 200 mm graphite	Standard process or AERB	Mold temperature: 1520 °C; Pouring temperature: 1520 °C; Withdrawal rate 6 mm/min
2 (E, P)	D = 180 mm quantity 7 graphite	AERB, d = 200 mm graphite	Modified process or IRBs	
3 (P)	-	PARB graphite	Modified process or PARB	
4 (P)	D = 180 mm quantity 7 molybdenum	AERB, d = 200 mm graphite	IRBs	
5 (P)	D = 180 mm quantity 14 graphite	AERB, d = 200 mm graphite	IRBs	

**Table 2:** Percentage reduction of primary dendrite arm spacing (PDAS) in casting for different manufacturing processes and solidification conditions.

Experiment Designation	Alloy	Geometry (Section Thickness)	Withdrawal Rate mm/min	Manufacturing Process	PDAS μm	Reduction of PDAS %	Reference
1 2	CMSX-4	Plate (6 mm)	6 6	Bridgman (AERB) Bridgman (IRB)	500 370	26	This work
3 4	CMSX-4	Blade (5 mm)	5 5	Bridgman (AERB) Bridgman (IRB)	480 360	25	[31]
5 6	CMSX-4	Blade (5 mm)	3 5	Bridgman (AERB) Bridgman (IRBs)	415 360	13	[31]
7 8		Rod (16 mm)	3.4 8.5	Bridgman LMC	380 250	34	[20]
9 10	Rene 4	IGT blade (10 mm)	2.5 5.1	Bridgman LMC	550 300	45	[16]
11 12	CMSX-4	IGT blade (10 mm)		Bridgman GCC	410 320	28	[43]
13 14	CMSX-4	Blade (5 mm)	2.5 2.5	Bridgman DWDS	445 299	33	[44]
15 16	CMSX-6	Rod (9 mm)	3 3	Bridgman DWDS	520 250	52	[45]
17 18	PWA1483	Blade (6 mm)	3 5	Bridgman FCBC	410 300	27	[46]
19	-	-	-	Bridgman (decrease of mold thickness)	-	8	[47]
